# CRISPR/Cas9-Mediated Overexpression of *HGF* Potentiates Tarim Red Deer Antler MSCs into Osteogenic Differentiation

**DOI:** 10.3390/ijms26178273

**Published:** 2025-08-26

**Authors:** Yujiao Qi, Xiaodong Jia, Chuan Lin, Wenxi Qian, Hong Chen, Di Fang, Chunmei Han

**Affiliations:** 1 College of Animal Science and Technology, Tarim University, Alar 843300, China; qiyujiao1128@163.com (Y.Q.); Jiaxiaodong1122@126.com (X.J.); linchuan0528@163.com (C.L.); qianwenxizj@163.com (W.Q.); difang@taru.edu.cn (D.F.); 2 Key Laboratory of Tarim Animal Husbandry Science and Technology, Xinjiang Production and Construction Corps, Alar 843300, China; 3 College of Life Sciences and Technology, Tarim University, Alar 843300, China; chenhong090588@163.com

**Keywords:** *HGF* gene, antler mesenchymal stem cells, CRISPR/Cas9, osteogenic differentiation, PI3K/Akt/mTOR pathway, MEK/ERK pathway

## Abstract

Previous studies conducted by our research groups have demonstrated that the HGF/c-Met signaling pathway promotes the proliferation and migration of MSCs in the antlers of Tarim red deer. However, the role and mechanism of this gene in the osteogenic differentiation of antler MSCs remain unclear. In this study, we used antler MSCs as experimental materials. CRISPR/Cas9 technology was employed to knock out the *HGF* gene, and lentivirus-mediated overexpression of the *HGF* gene was constructed in antler MSCs. Subsequently, antler MSCs were induced to undergo osteogenic differentiation in vitro. Alizarin Red staining was employed to identify calcium nodules, while the expression levels of various osteogenic differentiation marker genes were assessed using immunohistochemistry, RT-qPCR, and Western blotting techniques. The findings indicated that the *HGF* gene facilitates the osteogenic differentiation of antler MSCs. Analysis of genes associated with the PI3K/Akt and MEK/ERK signaling pathways demonstrated that in antler MSCs with *HGF* gene knockout, the expression levels of PI3K/Akt and MEK/ERK pathway genes were significantly downregulated on days 7 and 14 of osteogenic differentiation (*p* < 0.05). In contrast, antler MSCs with *HGF* gene overexpression exhibited a significant upregulation of the PI3K/Akt and MEK/ERK signaling pathways on days 4 and 6 of osteogenic differentiation (*p* < 0.01). These findings suggest that the *HGF* gene in antlers enhances the osteogenic differentiation of MSCs by activating the PI3K/Akt and MEK/ERK pathways.

## 1. Introduction

Deer antlers represent the only mammalian organ capable of regeneration, exhibiting a cell proliferation capacity that surpasses that of cancer cells during their rapid growth phase [1]. As a bony structure, the growth of antler velvet is facilitated through both intramembranous and endochondral osteogenesis, which occur via the proliferation and differentiation of antler stem cells (ASCs). ASCs are a recently identified class of stem cells, primarily categorized into Anterogenic Periosteum Cells (APCs), Pedicle Periosteum Cells (PPCs), and Reserve Mesenchyme Cells (RMCs) located within the apical growth center of the antler [2,3]. Jiyun Zhang et al. [4] observed antler mesenchymal stem cells (MSCs) using an electron microscope. The ultramicroscopic results indicated that antler MSCs possess a large cytosolic area, contain simple organelles, and exhibit mitochondrial and endoplasmic reticulum morphology characteristic of early naïve cell development. Studies have confirmed that ASCs exhibit features of both embryonic stem cells and ASCs and possess the ability to undergo multidirectional differentiation, allowing them to differentiate into various cell types, including osteoblasts, chondrocytes, adipocytes, and muscle cells [5,6].

Our group’s previous research on hepatocyte growth factor (HGF) in antler velvet revealed that *HGF* is highly expressed in the cartilage and bone tissues of Tarim red deer antlers. Furthermore, this gene promotes the proliferation of antler MSCs via the HGF/c-Met signaling pathway. However, the role of the *HGF* gene on the bone differentiation of antler MSCs remains unclear.

Several studies have indicated that the *HGF* gene plays a significant role in promoting bone differentiation [7,8]. However, in 2017, Kim et al. [9] inhibited the expression of the *HGF* gene and discovered that it could promote osteogenic differentiation in mouse cranial preosteoblasts and embryonic fibroblasts, thereby enhancing bone regeneration. Rachel and Kevin [10] proposed that the varying results regarding the influence of the *HGF* gene on bone differentiation may be attributed to the use of stem cells from different sources. Notably, no studies have reported on the effect of the *HGF* gene on the osteogenic differentiation of antler MSCs. In this study, we utilized CRISPR/Cas9 technology to edit antler MSCs for the first time, aiming to investigate the impact of HGF on their osteogenic differentiation. This research seeks to elucidate the mechanisms underlying the osteogenic differentiation of antler MSCs and to provide new insights for the clinical application of these cells. Furthermore, it aims to pave the way for advancements in stem cell regenerative medicine and tissue engineering.

## 2. Results and Analysis

### 2.1. Efficiency Assay of Knockdown and Overexpression of HGF Gene in Antler MSCs

**Figure 1:**
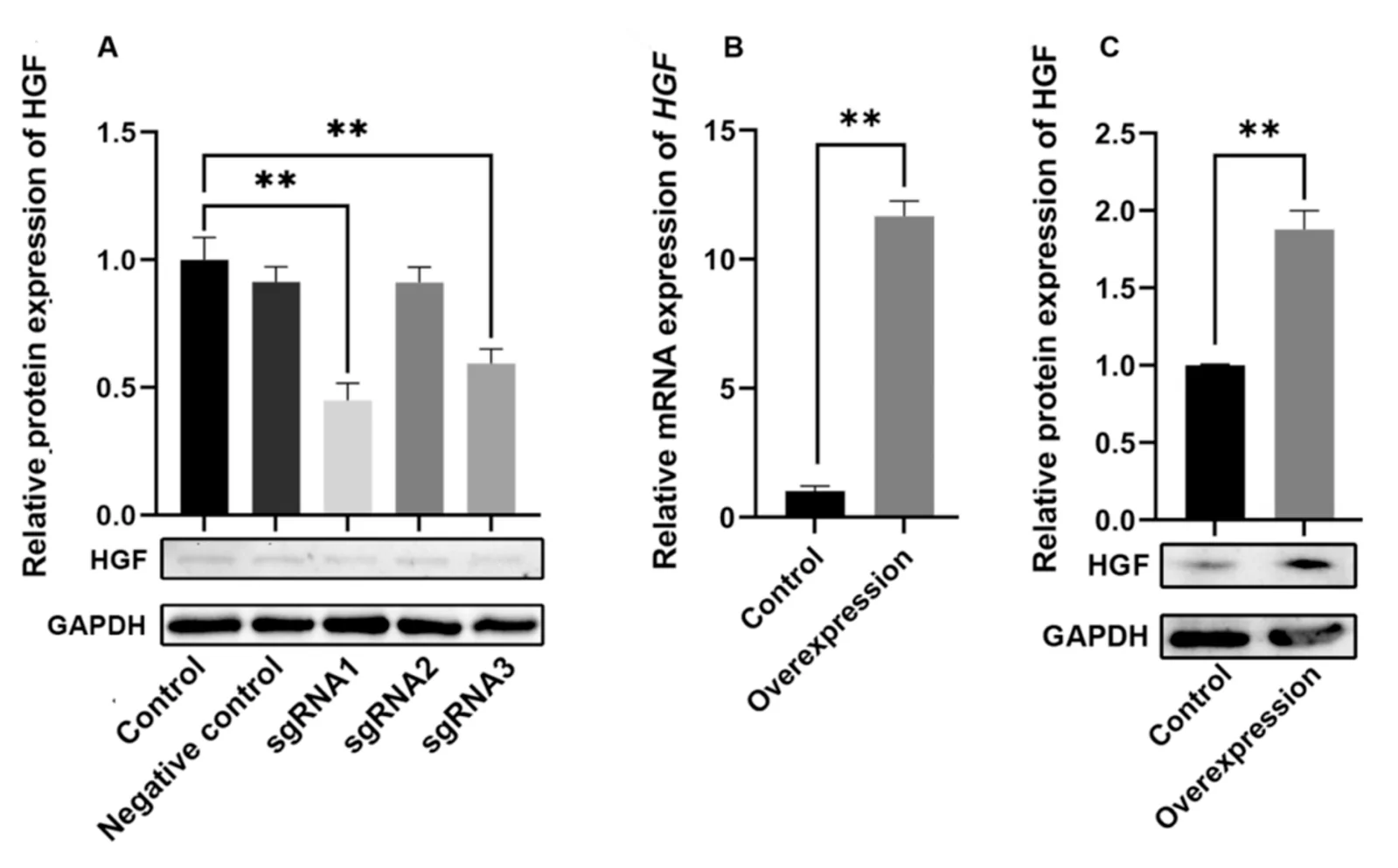
Experimental results of knockdown and overexpression of *HGF* gene. Note: (**A**): Western blotting was utilized to assess the efficiency of HGF knockdown guided by various sgRNAs. (**B**): RT-qPCR was employed to evaluate the efficiency of overexpressed HGF mRNA. (**C**): Western blotting was also conducted to determine the efficiency of overexpressed HGF. Data are presented as mean ± SD. ns: not significant; ** *p* < 0.01 compared with the control group.

The sgRNA1 group exhibited approximately a 55% reduction in HGF protein expression compared to the control group, demonstrating the highest knockdown efficiency (*p* < 0.01). Consequently, the sgRNA1 group was selected for subsequent experiments (see Figure 1A). RT-qPCR and Western Blotting analyses (Figure 1B,C) revealed that the overexpression efficiencies of the experimental groups were significantly higher than those of the control group (*p* < 0.01), confirming that antler MSCs effectively overexpressed the *HGF* gene.

### 2.2. Effect of HGF Gene on Osteogenic Differentiation of Antler MSCs

**Figure 2:**
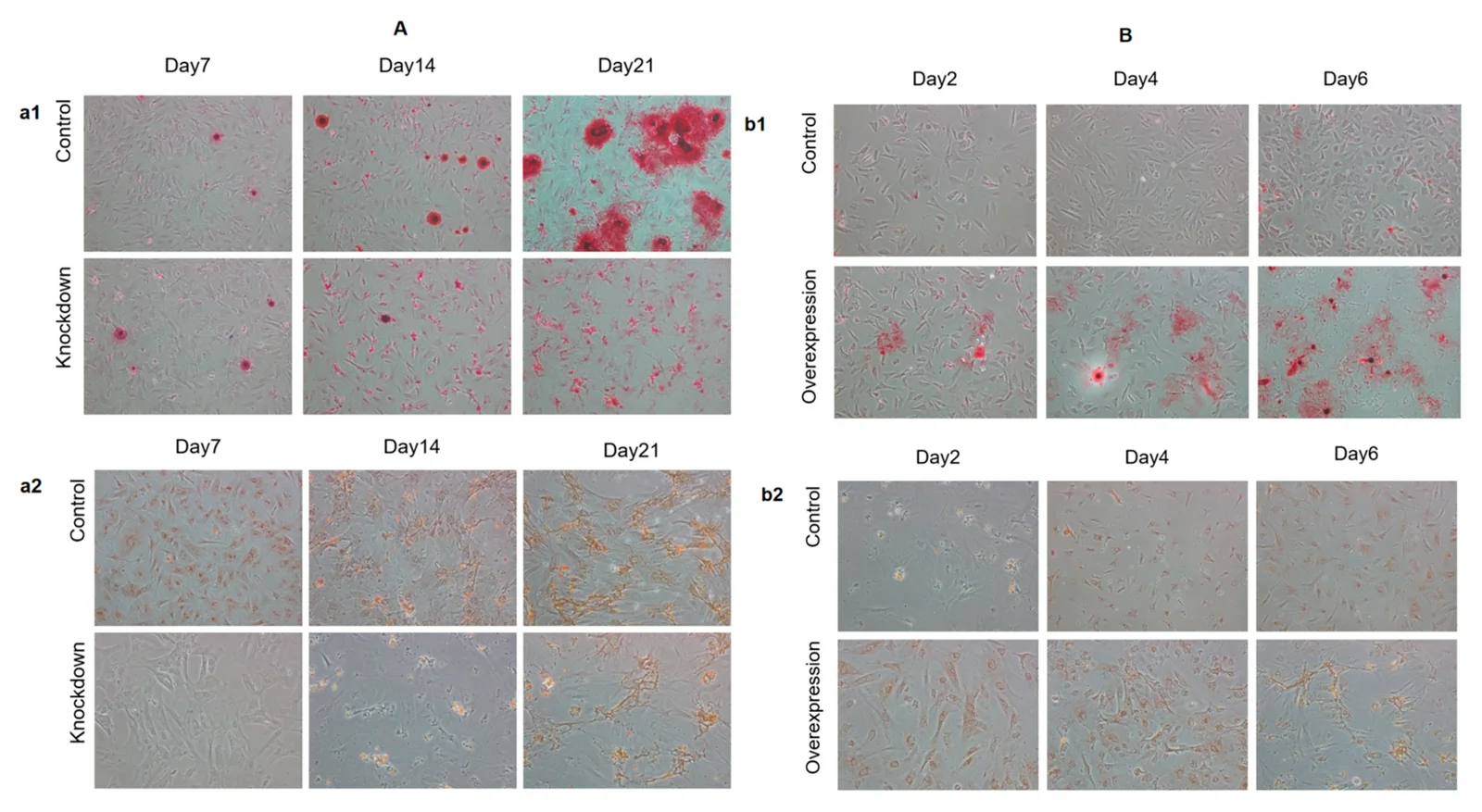

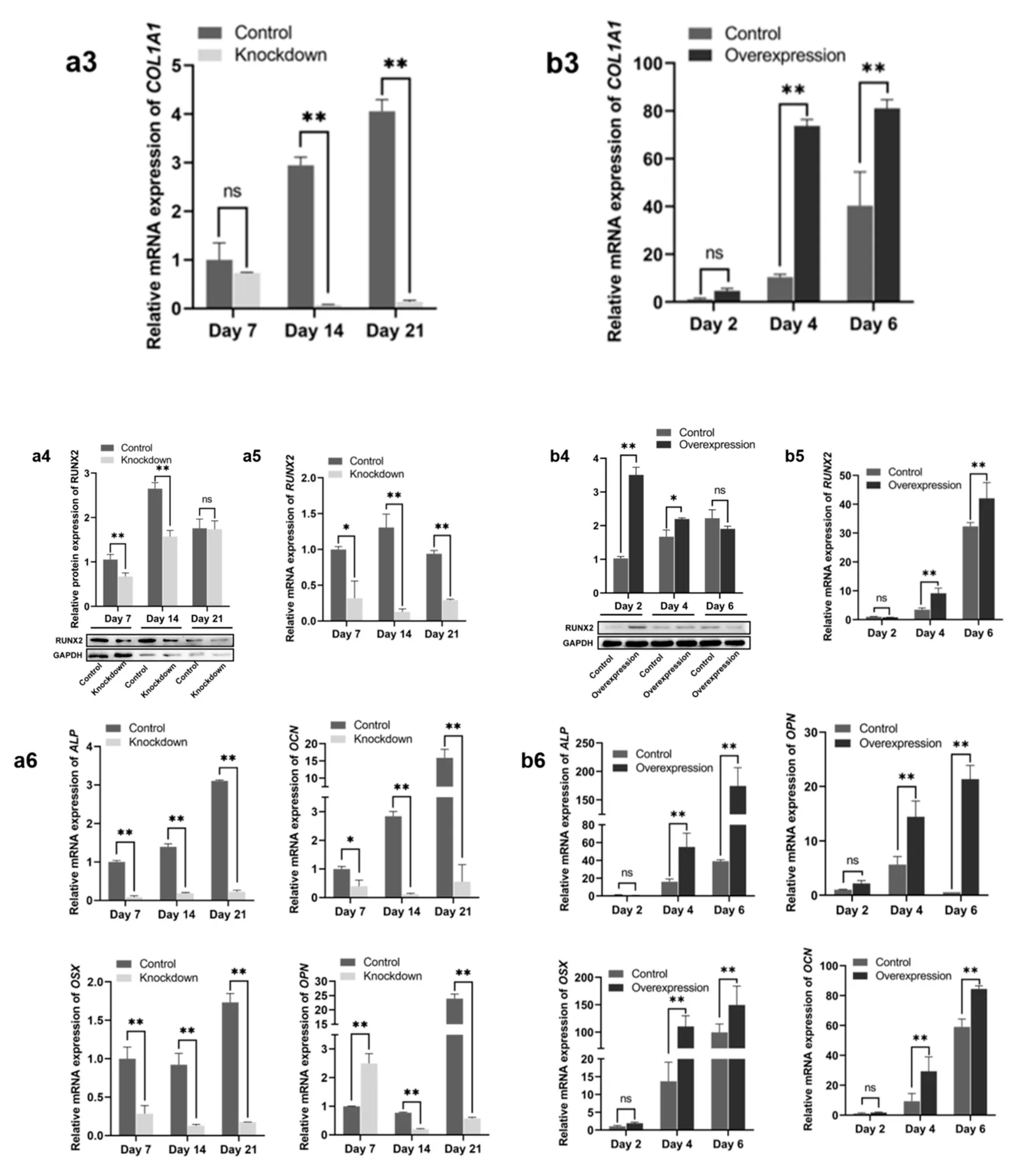
Experimental results on the effect of *HGF* gene on osteogenic differentiation of antler MSCs. Note: (**A**): antler MSCs HGF knockdown experimental group; (**B**): antler MSCs HGF overexpression experimental group. (**a1**): Results of alizarin red staining in the sgRNA1 group and control group at days 7, 14, and 21; (**a2**): Results of immunohistochemical analysis of COL1A1 protein expression in the sgRNA1 group and control group at days 7, 14, and 21; (**a3**): Results of RT-qPCR analysis of *COL1A1* gene expression in the sgRNA1 group and control group at days 7, 14, and 21; (**a4**): Results of RUNX2 protein Western Blotting analysis in the sgRNA1 group and control group at days 7, 14, and 21, where the positive signals appear brown; (**a5**): Results of *RUNX2* gene RT-qPCR analysis in the sgRNA1 group and control group at days 7, 14, and 21; (**a6**): Results of RT-qPCR analysis for *ALP*, *OCN*, *OSX*, and *OPN* genes in the sgRNA1 group and control group at days 7, 14, and 21. (**b1**): Results of alizarin red staining in the overexpression group and control group at days 2, 4, and 6; (**b2**): Results of immunohistochemical analysis of COL1A1 protein expression in the overexpression group and its control group at days 2, 4, and 6; (**b3**): Results of RT-qPCR analysis of *COL1A1* gene expression in the overexpression group and control group at days 2, 4, and 6; (**b4**): Results of RUNX2 protein Western Blotting analysis in the overexpression group and its control group at days 2, 4, and 6, where the positive signals appear brown; (**b5**):Results of RT-qPCR analysis of *RUNX2* gene expression in the overexpression group and its control group at days 2, 4, and 6; (**b6**): Results of RT-qPCR analysis of *ALP*, *OCN*, *OSX*, and *OPN* gene expression in the overexpression group and its control group at days 2, 4, and 6.Data are presented as mean ± SD. ns: not significant; * *p* < 0.05; ** *p* < 0.01 compared with the control group.

The results of alizarin red staining and immunohistochemical experiments of COL1A1 demonstrated a significant lag in the osteogenic differentiation process of antler MSCs following the knockdown of the antler *HGF* gene. Additionally, the proliferative capacity of the antler MSCs diminished after osteogenesis (see Figure 2(a1,a2,b1,b2)). Western blot analysis revealed that the expression of RUNX2 protein in the sgRNA1 group was significantly lower than that in the control group on both the 7th and 14th days (*p* < 0.01, see Figure 2(a4)). Conversely, the RUNX2 protein expression in the overexpression group was significantly higher than that of the control group on the 2nd day (*p* < 0.01) and also significantly elevated on the 4th day (*p* < 0.05, see Figure 2(b4)). RT-qPCR results indicated that the sgRNA1 group exhibited significantly lower levels of *COL1A1*, *RUNX2*, *ALP*, *OCN*, *OSX*, and *OPN* genes on the 14th and 21st days compared to the control group (*p* < 0.01, see Figure 2(a3,a5,a6)). In contrast, the mRNA expression levels of *COL1A1*, *RUNX2*, *ALP*, *OCN*, *OSX*, and *OPN* genes in the overexpression group were significantly higher than those in the control group on days 2 and 6 (*p* < 0.01, see Figure 2(b3,b5,b6)). Collectively, the results from alizarin red staining, immunohistochemistry, Western blotting, and RT-qPCR assays consistently indicated that the overexpression of *HGF* genes has a pronounced promoting effect on the osteogenic differentiation of antler MSCs.

### 2.3. Expression of PI3K/Akt and MEK/ERK Signaling Pathway Genes During sgRNA1 Group and Overexpression of Constitutive Bone Differentiation

**Figure 3:**
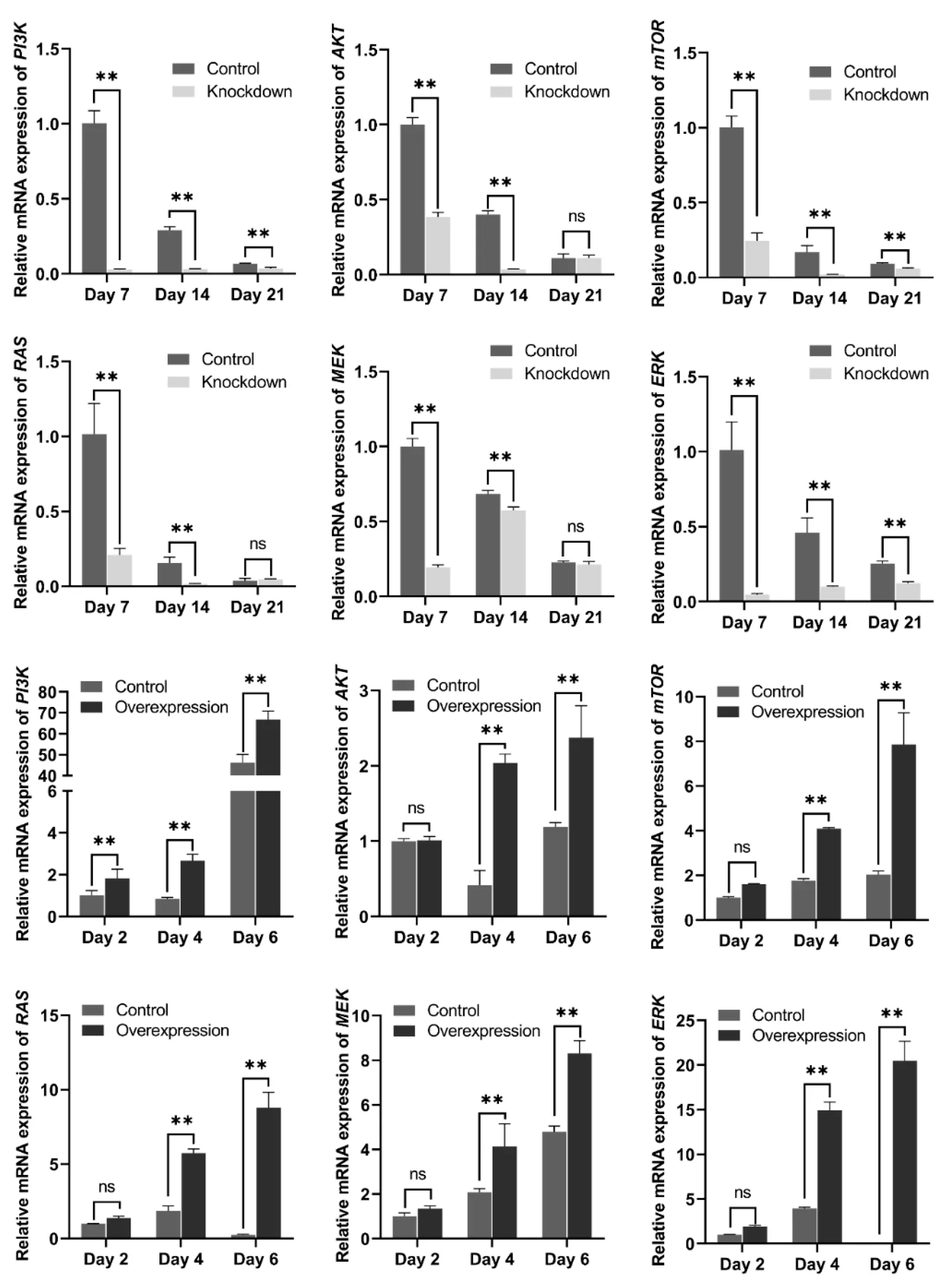
Relative mRNA expression of *PI3K*, *AKT*, *mTOR*, *RAS*, *MEK* and *ERK*. Note: Changes in the expression of PI3K/Akt and MEK/ERK signaling pathway genes in the sgRNA1 group and the control group at d 7, d 14, and d 21; Changes in the expression of PI3K/Akt and MEK/ERK signaling pathway genes in the overexpression group and the control group at d 2, d 4, and d 6. Data are presented as mean ± SD. ns: not significant; ** *p* < 0.01 compared with the control group.

The expression levels of the *PI3K*, *AKT*, *mTOR*, *RAS*, *MEK*, and *ERK* genes were assessed using RT-qPCR. The results demonstrated that these genes were significantly suppressed in the sgRNA1 group compared to the control group at days 7 and 14 of induced differentiation (*p* < 0.01). Conversely, in the overexpression group, the expression levels of *PI3K*, *AKT*, *mTOR*, *RAS*, *MEK*, and *ERK* genes were significantly upregulated (*p* < 0.01) at days 4 and 6 of induced differentiation (see Figure 3). These findings suggest that *HGF* genes enhance the PI3K/Akt and MEK/ERK signaling pathways.

## 3. Discussion

**Figure 4:**
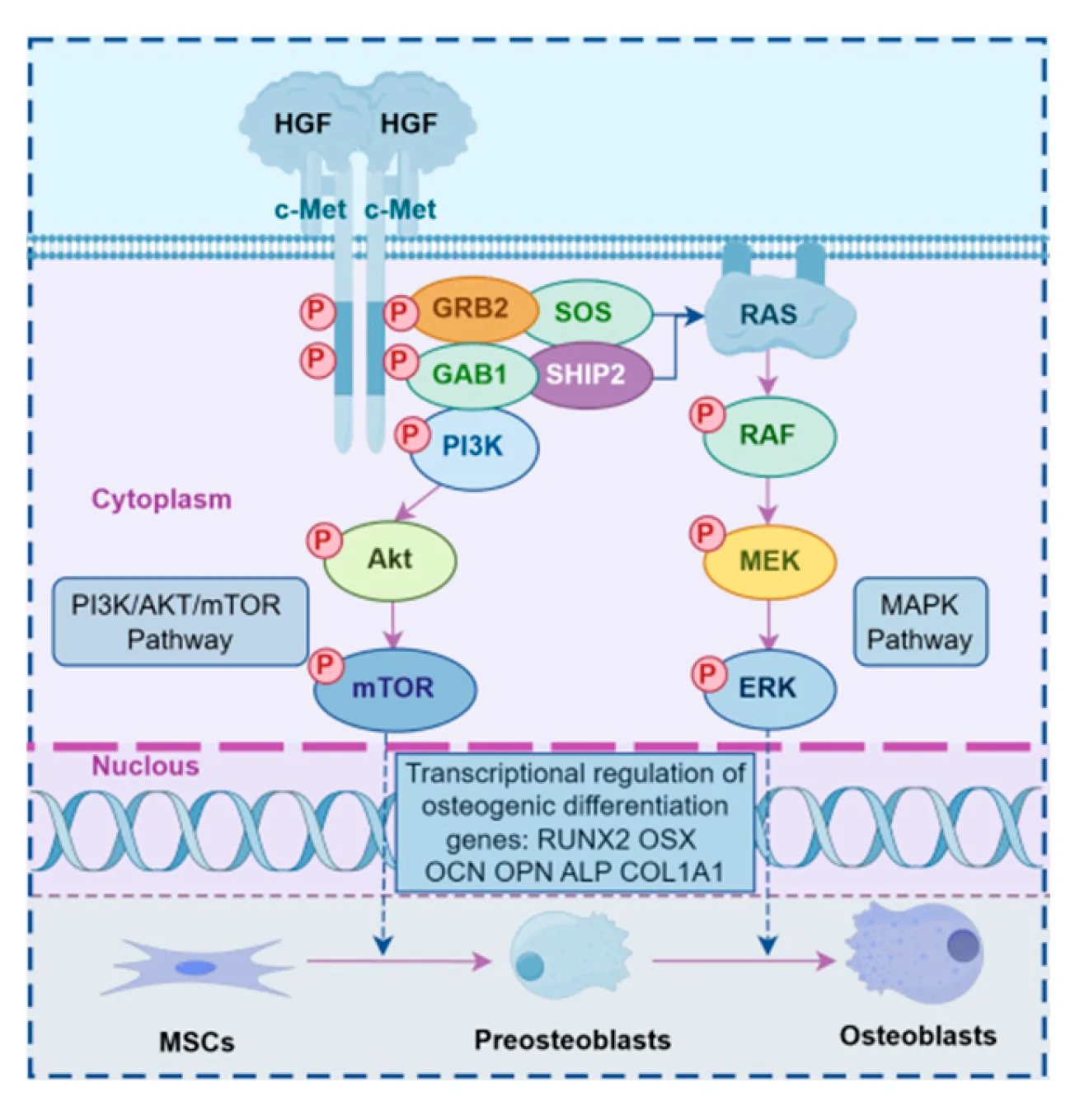
Schematic illustration of the proposed mechanism (by Figdraw). Note: Figure 4 Schematic diagram of the HGF/c-Met signaling pathways involved in osteogenic differentiation. Binding of HGF to its receptor c-Met activates downstream PI3K/AKT/mTOR and MAPK pathways, thereby regulating the transcription of osteogenic differentiation genes (*RUNX2*, *OSX*, *OCN*, *OPN*, *ALP*, *COL1A1*). P indicates phosphorylation; arrows indicate activation. Gene/protein abbreviations (e.g., HGF: hepatocyte growth factor; c-Met: mesenchymal-epithelial transition factor; PI3K: phosphatidylinositol 3-kinase; Akt: protein kinase B; mTOR: mammalian target of rapamycin; ERK: extracellular signal-regulated kinase; MEK: MAPK/ERK kinase; RAF: rapidly accelerated fibrosarcoma) are described in the text.

Han et al. [11] induced osteogenic differentiation of *P*_2_ MSCs derived from antlers using TGF-β1. They observed morphological changes in the cells on day 9 post-induction, with apoptosis noted by day 21. In the non-induced group, apoptosis began to manifest on day 28, and significant apoptosis was observed in both groups by day 35. This research confirmed that *P*_2_ MSCs from antlers are capable of undergoing osteogenic differentiation in vitro; however, the process is relatively slow. HGF binds to its receptor, c-Met, which leads to receptor dimerization and phosphorylation, transducing signals to intracellular downstream pathways such as the JAK/STAT3, PI3K/Akt/NF-κB, and MAPK pathways [12]. Aenlle et al. [7] induced human bone marrow MSCs to undergo cellular proliferation and found that the *HGF* gene promotes osteogenesis and osteoblastic differentiation by modulating the transcription of key osteogenic markers, including osteocalcin (OCN), osterix (OSX), and osteopontin (OPN). Shang et al. [13] investigated the role of rat femoral MSCs in bone defect repair by establishing a rat femoral defect model and found that the PI3K/Akt/mTOR pathway promotes the proliferation and differentiation of both osteoblasts and osteoclasts. In 2018, Lu et al. [14] confirmed that the osteogenic activity of RUNX2 and OCN may be enhanced through phosphorylation-mediated modification of the MAPK pathway by knocking out the *PaX2* gene in bone marrow MSCs isolated and cultured from 4–6-week-old mice. This modification further promotes the interaction between RUNX2 and OCN. Additionally, the activation of the PI3K/Akt signaling pathway has been identified as a key regulatory cascade for osteogenic differentiation, and its activation has been shown to induce enhanced osteogenic activity [15].In the present study, we determined that the antler *HGF* gene regulates the differentiation of antler MSCs into bone through the RAS/MEK/ERK and PI3K/AKT/mTOR signaling pathways. HGF binds extracellularly to the c-Met ligand, promoting the phosphorylation of GRB2, GAB1, PI3K, and SHIP2. Subsequently, GRB2 binds to SOS, which activates the RAS protein, initiating the RAF-containing MEK and ERK proteins that activate the MAPK signaling pathway. Concurrently, c-Met activates AKT through PI3K, and AKT further activates mTOR [16]. These two signaling pathways work synergistically to promote the transcription of osteogenic differentiation marker genes, such as *RunX2*, *OSX*, *OCN*, *OPN*, *ALP*, and *COL1A1*, in the nucleus, thereby facilitating the osteogenic differentiation of stem cells (see Figure 4).

## 4. Materials and Methods

### 4.1. Material

In this experiment, three healthy adult male Tarim mare deer, bred at the Tarim Primary Breeding Farm of the 31st Division in Xinjiang, China, were selected. Systematically characterized *P*_2_ generation antler MSCs and MSCs overexpressing antler *HGF* genes were sourced from the Tarim Key Laboratory of Animal Husbandry Science and Technology (China). These samples were collected at the transition from the saw of antler growth to the rapid growth period of 53 days 

### 4.2. Methods

#### 4.2.1. CRISPR/Cas9 Knockdown of *HGF* Gene in Deer Antler MSCs

**Figure 5:**
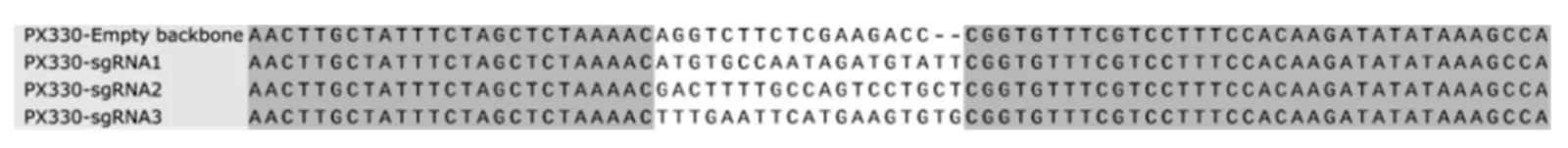
Plasmid Sequencing Results of PX330-sgRNA1, PX330-sgRNA2, and PX330-sgRNA3. Note: Gray background: pX330 plasmid backbone; white background: sgRNA sequence.

The mRNA sequence of the Tarim red deer HGF (XM_043873082.1) served as a template for the selection of sgRNA sequences. Using Crispor https://insectomics.net/CRISPR/manual/ (accessed on 7 September 2023), the three highest-scoring sgRNA sequences were chosen and cloned into the pX330 vector, which was generously provided by the Institute of Animal Husbandry Science, Xinjiang Academy of Animal Husbandry. The culture medium was prepared as DMEM-low glucose complete medium (C11885500BT, Gibco Laboratories, Grand Island, NY, USA), supplemented with 10% fetal bovine serum (10099141, Gibco Laboratories, Grand Island, NY, USA) and antibiotics (100 U/mL penicillin and 100 μg/mL streptomycin, both sourced from BL505A, Shandong White Shark Biotech Co. Ltd., Tengzhou, China). Cells were inoculated at a density of 1×10^5^ cells per well in six-well plates and incubated at 37 °C in a 5% CO_2_ cell culture incubator (BPN-30CW (UV), Shanghai Yiheng Technology Co., Ltd., Shanghai, China). The medium was changed routinely every two days, based on cell growth observations. Cell density was monitored under a microscope (DMi1, Leica Microsystems GmbH, Wetzlar, Germany), and when it reached 70%, transfection was conducted following the instructions for the TransIntro^®^EL transfection reagent (FT201-01, Beijing Total Gold Biological Co., Ltd., Beijing, China). The medium used for transfection was consistent across all experimental groups. Three CRISPR/Cas9 plasmids targeting the *HGF* gene were successfully constructed and designated as pX330-sgRNA5, pX330-sgRNA2, and pX330-sgRNA3. The sequencing results are presented in Figure 5. The experiment was divided into five groups: the sgRNA1 group (cells transfected with the pX330-sgRNA1 plasmid), the sgRNA2 group (cells transfected with the pX330-sgRNA2 plasmid), the sgRNA3 group (cells transfected with the pX330-sgRNA3 plasmid), the negative control group (cells transfected with the empty pX330 plasmid), and the control group (antler velvet antler MSCs serving as a blank control without transfection).

#### 4.2.2. Tissue RNA and Protein Extraction

After 72 h of transfection, mRNA and protein were extracted from antler tissues using the Trizol method (Invitrogen, 15596018, Carlsbad, CA, USA) and a chemical lysis method, respectively, for antler MSCs with knockdown and overexpression of the *HGF* gene.

#### 4.2.3. Real-Time Fluorescence Quantitative PCR

Primer Premier 6.0 software was utilized to design primers for osteogenic differentiation marker genes, *GAPDH*, and genes associated with the PI3K/Akt/mTOR and MEK/ERK pathways. RT-qPCR primers are listed in Table 1. The RT-qPCR was conducted using Newbay Bio HyperScript lll RT SuperMix for qPCR with the gDNA Remover kit (R202-02, EnzyArtisan, Shanghai, China) to assess changes in the expression levels of osteogenic differentiation marker genes across different groups using the SYBR Green method. The reaction conditions were as follows: pre-transformation at 95 °C for 3 s; denaturation at 95 °C for 10 s; annealing at 56–61 °C for 10 s; extension at 72 °C for 15 s; and a final extension at 72 °C for 5 min, comprising a total of 30 cycles.

#### 4.2.4. Inducible Expression of pX330-HGF Protein

*P*_2_ generation antler MSCs were seeded in six-well plates at a density of 1.5 × 10^5^ cells and transfected for 72 h. When the cell density reached 70%, total cellular proteins were extracted separately, quantified, and subjected to SDS-PAGE electrophoresis. The proteins were then transferred to PVDF membranes (IPVH00010, Merck Millipore, Darmstadt, DE, Germany) and blocked with TBST containing 5% skimmed milk powder for 1 h. The primary antibody was incubated overnight at 4 °C in a refrigerator, followed by incubation with a sheep anti-rabbit secondary antibody at room temperature (dilution ratio of 1:15,000, LF102, Shanghai Yase Pharmaceutical Science and Technology Co., Ltd., Shanghai, China). Protein expression was detected by adding ECL chemiluminescent solution (E1050, Mimus Biotechnology Co., Ltd., Xi’an, China) and analyzed in grayscale using ImageJ (1.52u) software. The plasmid with the highest knockdown efficiency was selected for subsequent experiments.

#### 4.2.5. In Vitro Induction of Osteogenic Differentiation by Antler MSCs

The sgRNA1 group, along with its control group, and the overexpression group, along with its control group, were cultured in vitro for 48 h. Following this incubation period, the medium was replaced with osteogenic differentiation medium, which included 10% FBS, 1% PS, 10 mM sodium β-glycerophosphate (Beijing Solepol Science and Technology Co., Ltd., G8100, Beijing, China), 50 μg/mL ascorbic acid (A7506, Sigma-Aldrich, St. Louis, MO, USA), and 10 nM dexamethasone (D6950, Beijing Solepol Technology Co., Ltd., Beijing, China) in DMEM-low glucose.

#### 4.2.6. Alizarin Red Staining to Identify the Effects of Osteogenic Differentiation

Cells from the antler MSCs in the sgRNA1 group and the control group were stained with alizarin red on days 7, 14, and 21 using the Alizarin Red S Staining Quantitative Detection Kit (G1450, Beijing Solebo Technology Co., Ltd., Beijing, China). On day 7 of induced differentiation, the antler MSCs in the overexpression group and the control group exhibited excessive ossification and signs of apoptosis. Consequently, cells from both the overexpression and control groups were stained with alizarin red, focusing on cells from the induced differentiation at days 2, 4, and 6.

#### 4.2.7. Immunohistochemical Identification of Osteogenic Differentiation Effects

COL1A1 immunohistochemistry and RT-qPCR were conducted on the 7th, 14th, and 21st days of induced differentiation in the sgRNA1 group and the control group. Similarly, COL1A1 immunohistochemistry and RT-qPCR were performed on the 2nd, 4th, and 6th days of induced differentiation in the overexpression group and the control group. The 6 μm frozen sections, stored at −80 °C, were thawed at room temperature for 30 min. Subsequently, they were incubated with acetone (126, Tianjin Zhiyuan, Tianjin, China) at 4 °C for 20 min, permeabilized with 0.1% Triton X-100 (1610407, Bio-Rad, Hercules, CA, USA) for 30 min, and blocked with 10% goat serum for 1 h. The sections were then incubated overnight at 4 °C with a rabbit anti-COL1A1 polyclonal antibody (1:200 dilution). The following day, a goat anti-rabbit IgG polymer (1:100 dilution) from the PV-9003, Beijing Zhongshan Jinqiao Biotechnology Co., Ltd., Beijing, China) and Rabbit 2-Step Kit (PV-9001, Zhongshan Golden Bridge Biotechnology Co., Ltd., Beijing, China) was added dropwise and incubated for 20 min. DAB staining was conducted for observation using an inverted microscope (Eclipse Ti, Nikon Corporation, Tokyo, Japan), and gray scale analysis was performed using ImageJ (version 1.52u) software.

### 4.3. Statistical Analysis

Data were analyzed using SPSS 26.0, GraphPad Prism 5, and ImageJ (version 1.52u). For normally distributed data, measures are expressed as mean ± standard deviation Comparisons between two groups were conducted using the *t*-test, while comparisons of means among three or more groups were performed using one-way ANOVA. Percentage differences between two groups were analyzed using the chi-square test. Asterisks indicate statistical significance, with ‘**’ representing *p* < 0.01 and ‘*’ representing *p* < 0.05.

## 5. Conclusions

The *HGF* gene from the Tarim red deer antlers promotes the osteogenic differentiation of antler MSCs by up-regulating the PI3K/Akt/mTOR and MEK/ERK signaling pathways. Additionally, CRISPR/Cas9 technology was successfully used to generate HGF-deficient anterior MSCs.

## Figures and Tables

**Table 1:** Sequences of the Primers.

Gene	Sequence(5′-3′)	Annealing Temperature/℃	Fragment Length/bp	Accession Numbe
*GAPDH*	F: TGTTTGTGATGGGCGTGAACCA R: ATGGCGTGGACAGTGGTCATAA	61	154	NM_001034034.2
*ALP*	F:ACATCGAGGTGATCATGGGC R:GATCAGTGCGGTTCCAGACA	60	183	XM_043909115.1
*COL1A1*	F:CCAATGGCGCTCCTGGTATT R:ACCAGGTTCACCGCTGTTAC	60	116	XM_043904209.1
*OCN*	F:GAAGAGACTCAGGCGCTACC R:GCTAGCTCGTCACAGTCAGG	60	114	XM_043878504.1
*OSX*	F:TCCCTGCTTGAGGAGGAAG R:GGCTTCTTTGTGCCTGCTTT	59	131	XM_043882724.1
*OPN*	F:CCTCCGCCCTTCCAGTTAAA R:CTGCTTCTGAGATGGGTCAGG	60	116	XM_043871261.1
*RUNX2*	F:CAAGGTGGTAGCCCTTGGAG R:AACAGCAGAGGCATTTCGGA	60	103	XM_043907628.1
*PI3K*	F:GCAGACTGGAGGGAGGTGA R:TCCGCAAGGTCAAAGTGTAA	59	280	XM_043876754.1
*AKT*	F:CGCACCGCTCCAAAGAAA R:ACGGCTGCACGTAGACACC	59	199	XM_043898476.1
*mTOR*	F:CGTCTGTCGGTGGTCTTTG R:CGGAGGTTCCCATCTTTC	57	169	XM_043915272.1
*RAS*	F:CCAGTGGAGGATGACGAG R:CCCATAGGCAGGAGTGAA	56	183	XM_043924996.1
*MEK*	F:CGAAAGGCAAGAAGCGAAAC R:TCCACGATGGGCTCCAGGTC	61	163	XM_043905687.1
*ERK*	F:AGACGCAACACCTCAGCA R:GCCAAGCCAAAGTCACAGA	59	163	XM_043901129.1

## Data Availability

The original contributions presented in this study are included in the article. Further inquiries can be directed to the corresponding author.
